# The Genome of the Softshell Clam *Mya arenaria* and the Evolution of Apoptosis

**DOI:** 10.1093/gbe/evaa143

**Published:** 2020-07-11

**Authors:** David C Plachetzki, M Sabrina Pankey, Matthew D MacManes, Michael P Lesser, Charles W Walker

**Affiliations:** e1 Molecular, Cellular and Biomedical Sciences, University of New Hampshire; e2 School of Marine Science and Ocean Engineering, University of New Hampshire

**Keywords:** apoptosis, gene family evolution, *Mya*, softshell clam, Ecdysozoa

## Abstract

Apoptosis is a fundamental feature of multicellular animals and is best understood in mammals, flies, and nematodes, with the invertebrate models being thought to represent a condition of ancestral simplicity. However, the existence of a leukemia-like cancer in the softshell clam *Mya arenaria* provides an opportunity to re-evaluate the evolution of the genetic machinery of apoptosis. Here, we report the whole-genome sequence for *M. arenaria* which we leverage with existing data to test evolutionary hypotheses on the origins of apoptosis in animals. We show that the ancestral bilaterian p53 locus, a master regulator of apoptosis, possessed a complex domain structure, in contrast to that of extant ecdysozoan p53s. Further, ecdysozoan taxa, but not chordates or lophotrochozoans like *M. arenaria*, show a widespread reduction in apoptosis gene copy number. Finally, phylogenetic exploration of apoptosis gene copy number reveals a striking linkage with p53 domain complexity across species. Our results challenge the current understanding of the evolution of apoptosis and highlight the ancestral complexity of the bilaterian apoptotic tool kit and its subsequent dismantlement during the ecdysozoan radiation.

## Introduction

Programed cell death, apoptosis, is a fundamental feature of multicellular animals where it is essential during development, pathogen response, and suppression of carcinogenesis. Therefore, understanding the evolution of the intricate cellular machinery of apoptosis is essential for our interpretation of how complexity in multicellular animals arose. Among animals, mammals are considered the pinnacle of apoptotic complexity and initiate apoptosis using multiple, distinct, p53-mediated pathways. Apoptosis may result from the binding of extracellular death ligands through an extrinsic death receptor-driven pathway (Srinivasula et al. 1996) or may be initiated in response to DNA damage by an intrinsic transcriptional pathway (Vogelstein and Kinzler 1992). Alternatively, internal or external stress can exert proapoptotic effects directly on mitochondria without additional transcription (Mihara et al. 2003; Walker et al. 2011). Working together or separately, each of these pathways leads to formation of the apoptosome, mitochondrial catastrophe, and destruction of the nuclear genome (Aubrey et al. 2018).

Apoptosis is widespread in bilaterian animals, but outside of vertebrates it is best understood in the ecdysozoan animal models *Drosophila melanogaster* and *Caenorhabditis elegans* (Zou et al. 1997; Steller 2008). However, several lines of evidence suggest that the composition and function of apoptosis pathways in these taxa may be a derived condition shaped by their unique life histories. For instance, unlike most animal lineages, the adults of both ecdysozoan animal models largely lack mitotic somatic cells, limiting the risk of uncontrolled cell division, one of the major functions of apoptosis in mammals. Apoptosis is most important in development and patterning in the short-lived ecdysozoan animals and, except for defective germ cells in adults, is irrelevant for removing damaged or malignant cells (Bangs and White 2000; Mishra et al. 2018). For these reasons, our understanding of the evolution of apoptosis may be biased by the life histories of two of the best-studied invertebrate models. Indeed, the current paradigm portrays the mode of apoptosis in *D. melanogaster* and *C. elegans* as representative of an ancestral condition, from which mammalian-grade apoptotic complexity emerged (Lu et al. 2009; Belyi et al. 2010).

However, many invertebrate species possess long-lived adult stages and persistent somatic stem cell populations (Pearson and Sanchez Alvarado 2010). One example is the softshell clam, *Mya arenaria*, which is found worldwide in marine soft-bottom ecosystems. In nature, *M. arenaria* is known to develop a potentially transmissible leukemia that has been described as an alternative invertebrate model for cancer (Walker et al. 2011; Fernandez Robledo et al. 2019). Here, we integrate comparative analyses of the metazoan p53 gene family, a master regulator of apoptosis (Vogelstein and Kinzler 1992), and a suite of functionally related genes involved in apoptosis using genome sequence data from a suite of phylogenetically informative taxa including the genome sequence of *M. arenaria*, which we report here. Unlike p53 loci from *D. melanogaster* and *C. elegans*, we show that p53 loci from *M. arenaria* and other lophotrochozoans (mollusks, annelids, and their relatives) possess a complex p53 domain structure reminiscent of the mammalian p53, p63, and p73 paralogs. In addition, phylogenetic comparative analyses of the distributions of apoptotic gene orthologs indicates that a complex apoptotic genetic repertoire was present in the last common ancestor of bilaterian animals and that this complexity was retained in mammals and lophotrochozoans but lost in early ecdysozoan ancestors. Our analyses also demonstrate a striking relationship between the richness of the apoptosis genetic repertoire and p53 domain complexity across the metazoan tree suggesting a possible adaptive mechanism for apoptotic complexity reduction in ecdysozoans.

Together our findings force a reevaluation of the current view of the evolution of the p53 gene family and the genomic landscape of p53-mediated apoptosis that suggests that the regulation of apoptosis had likely achieved mammalian-grade complexity by the time of the bilaterian ancestor and is conserved in lineages including mollusks and mammals, but has been subsequently lost in short-lived ecdysozoan lineages including *D. melanogaster* and *C. elegans.* Our phylogenetic comparative analyses also suggest that alterations in p53 domain structure may have helped precipitate the widespread genomic alterations in apoptotic gene copy number that we observe during the bilaterian radiation.

## Results

### A Draft Genome of the Softshell Clam *Mya arenaria*

**Fig. 1.**
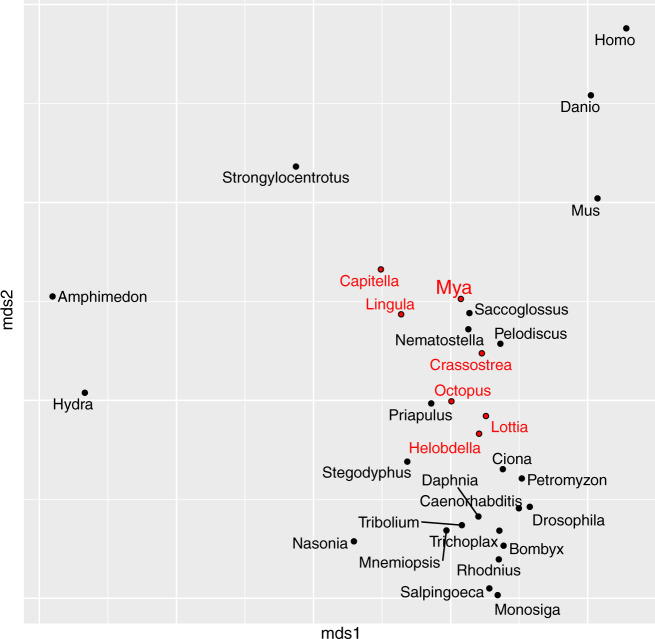
The *Mya arenaria* protein-coding genome complement is similar to other lophotrochozoans. Multidimensional scaling plot of showing ordination of genomic similarity between high-quality metazoan genomes including *M. arenaria*. All lophotrochozoan genomes are shown in red.

The draft assembly of the *M. arenaria* genome based on paired-end and mate pair reads yielded an assembly of 1.19 Gb and an N50 of 47 kb. The final assembly contained 76% complete BUSCOs, with 13% of the genes duplicated and 17% missing. Together these statistics indicate that our assembly of the *M. arenaria* genome is highly contiguous and nonredundant. RepeatMasker masked 35% of the genome as repetitive. LINE1 and LTR elements alone constituted 21% of the repeats. Coding gene content of the *M. arenaria* genome is similar to that of other lophotrochozoans (fig. 1).

### p53 Phylogeny Demonstrates an Early Origin for Complex Domain Structures

**Fig. 2.**
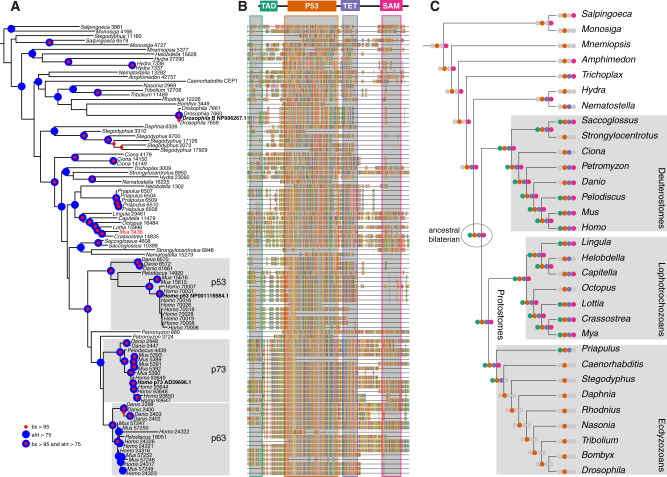
The evolutionary history and functional diversification of choanozoan p53s. (*A*) Phylogeny of choanozoan p53 sequences obtained from whole-genome sequences. Maximum likelihood tree was estimated under the best fit VT+F+R6 model (Quang le et al. 2008). Support is given by aLRT (Anisimova and Gascuel 2006) and ultrafast bootstrapping (Hoang et al. 2018). Vertebrates p53, p63, and p73 clades are indicated. Lophotrochozoan sequences are consistently more similar to mammalian sequences and reside on relatively short branches in contrast to ecdyzozoan sequences which reside on characteristically long branches. (*B*) Multiple sequence alignment showing the domain structure of p53 gene family sequences on the phylogeny. Approximate locations of the TAD, p53, TET, and SAM domains are shown. (*C*) Phylogeny of the whole-genome taxa utilized in this study. Clades of interest include the deuterostomes including mammals, lophotrochozoans including *Mya arenaria*, and ecdysozoans including *Drosophila melanogaster* and *Caenorhabditis elegans*. Dollo parsimony was used to map the evolutionary histories of the individual p53 gene family domains onto the phylogeny. This analysis portrays an increase in domain complexity reaching its peak at the bilaterian ancestor. Much of this domain complexity is retained in deuterostomes and lophotrochozoans but has been lost in nonpriapulid ecdysozoans. Colors are as shown in (*B*).

In order to understand the evolutionary history of metazoan p53, we conducted phylogenetic analyses using a selection of whole-genome data sets, including *M. arenaria*, representing all of the major lineages of animals including their choanoflagellate sisters. Our phylogenetic analyses of p53 indicate an early origin of the gene family that predates Metazoa (Belyi et al. 2010; Rutkowski et al. 2010) (fig. 2*A*). p53/p63/p73 loci were recovered from all protein models examined including those of choanoflagellates, ctenophores, sponges, cnidarians, placozoans, and all bilaterians. p53 sequences from all ecdysozoan species, with the exception of the priapulid *Priapulus caudatus*, were positioned toward the base of the tree and resided on characteristically long branches. We note the significant possibility that our reconstruction of choanozoan p53 phylogeny is hindered by long-branch attraction (Bergsten 2005) of short, highly divergent ecdysozoan sequences to the base of the tree. Therefore, the placement of most ecdysozoan p53s in the choanozoan tree (except *Priapulus*) is likely a systematic artifact. Indeed, the placement of the nonpriapulid ecdysozoan p53 sequences is poorly supported and several features of these sequences are degenerate and/or highly divergent (fig. 2*A*).

The diversity of domain complements across the p53 superfamily genes elicits a range of functions from the transcriptional activation of proapoptotic expression profiles to development (Vogelstein and Kinzler 1992). In order to understand the evolutionary history and functional diversification of the p53 gene family, we determined the domain architecture for each sequence included in figure 2*A* using the Pfam database (El-Gebali et al. 2019) and examined these character histories on the species tree using the principle of Dollo parsimony (fig. 2*C*) in which traits (e.g., domains) may evolve once and may be lost, but may not reoriginate in descendant branches (Farris 1977). This analysis highlights contrasting domain richness among protostome p53 loci and shows that all nonpriapulid ecdysozoan p53 sequences, as in *Drosophila* or *Caenorhabditis*, include only the p53 DNA binding domain. None of the other protein–protein interaction domains presents in other bilaterian sequences including the transactivation domain (TAD) (Lee et al. 2010), the tetramerization domain (TET) (Joerger et al. 2014) or the sterile alfa motif domains (SAM) (Ou et al. 2007) are present in nonpriapulid ecdysozoan p53s. Conversely, all lophotrochozoan p53 loci, including the p53 locus from *M. arenaria*, show a greater domain complexity than any ecdysozoan sequence, making lophotrochozoan p53s more akin to their vertebrate homologs. Here, lophotrochozoan p53s are especially rich in TAD, TET, and SAM domains and approximate the additive domain complexity of vertebrate p53, p63, and p73 paralogs (Aberg et al. 2017). Our results indicate that the ancestral bilaterian p53 gene(s) exhibited the full complement of p53 domain complexity observed in extant taxa, with subsequent alterations being due to domain loss largely confined to the ecdysozoan lineages that diverged after the split with priapulids (fig. 2*C*).

### Apoptosis Gene Enrichment Analysis Indicates Depletion of Apoptosis Pathways in Ecdysozoans but Not in Lophotrochozoans

**Fig. 3.**
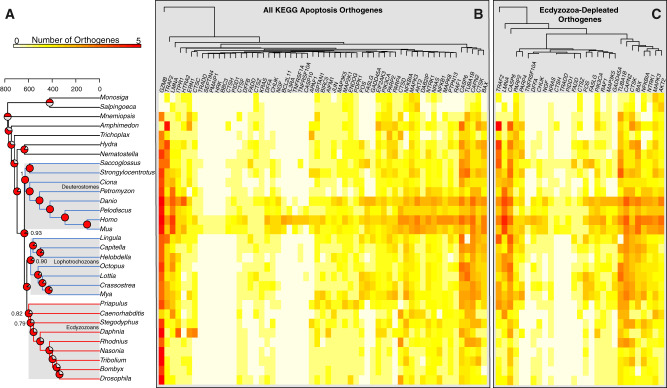
The evolutionary history of apoptotic genes as revealed by ortholog enrichment analysis. (*A*) Time calibrated phylogeny of the whole-genome taxa included in the study. Pie charts indicate additive gene totals from apoptosis orthogroups that are significantly depleted in ecdysozoans as revealed by Dollo parsimony analyses. (*B*) Heatmap showing gene counts across all 57 orthogroups derived from the analysis of KEGG apoptosis genes. (*C*) The subset of genes that is significantly depleted in ecdysozoans (red branches) as compared with deuterostomes and lophotrochozoans (blue branches). The cells in the heatmaps are ordered based on hierarchical clustering, shown at top.

In vertebrates, p53 exerts its modulation of apoptosis through intrinsic damage-dependent, extrinsic ligand-driven, and direct mitochondrial routes via interactions with a set of proteins that interface with diverse cellular pathways. In order to understand the genomic complement of these other apoptosis proteins in *M. arenaria* and across Metazoa, we conducted an orthology analysis that placed individual sequences from the protein models of each taxon into orthogroups (Emms and Kelly 2015). We then identified the orthogroups associated with the human sequences represented by each of 137 curated loci from the mammalian KEGG apoptosis gene set (Kanehisa et al. 2019) and tabulated the presence or absence of genes for all other taxa for each of the resulting 59 apoptosis orthogroups (fig. 3*A* and *B*). A proportion of KEGG apoptosis loci either eluded orthology assignment due to short sequence length (56/137 KEGG loci) or were homologized to the same orthogroup in these analyses (22 of remaining 81 KEGG loci). Of the 59 KEGG apoptosis orthogroups recovered in our analysis, 29 (49%) showed significant enrichment in gene number among nonecdysozoan bilaterians (e.g., deuterostomes and lophotrochozoans), whereas no apoptosis orthogroups were enriched in the set of ecdysozoan genomes compared with nonecdysozoan bilaterians (FDR < 0.1) (fig. 3*C* and supplementary table 1, Supplementary Material online).

In order to establish that this result is driven by ecdysozoan-specific gene loss and not vertebrate- or lophotrochozoan-specific duplicative gene gain, we conducted two additional analyses. First, we examined additional statistical contrasts of gene gain/loss by comparing only lophotrochozoans and ecdysozoans (supplementary fig. 1, Supplementary Material online), which showed a similar pattern of ecdysozoan gene depletion as in figure 3*C*, suggesting that vertebrate-specific gene duplications do not drive our result. Second, we examined the evolutionary histories of each individual apoptosis orthogroup across the metazoan tree using Dollo parsimony (fig. 3*A*). These analyses show that a nearly complete apoptosis gene set, based on human KEGG pathway, was present in the bilaterian ancestor and that the significant differences in the distributions of these genes observed between ecdysozoan and nonecdysozoan genomes is largely due to orthogroup loss in the ecdysozoan lineage, whereas deuterostomes and lophotrochozoan genomes have retained largely intact ancestral apoptotic genetic repertoires (fig. 3*A*).

**Fig. 4.**
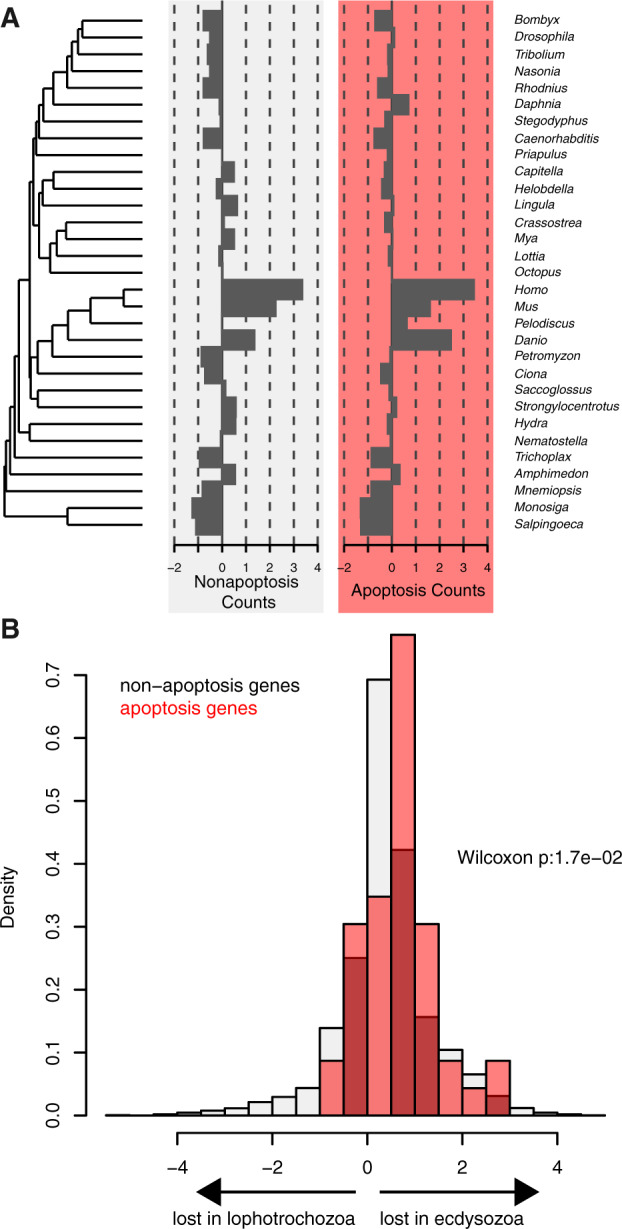
The relationship between genome size and number of apoptosis genes across choanozoan phylogeny. We observed marginal phylogenetic signal for genome size when analyzed on the phylogeny (*K* = 0.65; λ=0.71). (*A*) This relationship is visible when the numbers of genes recovered in our orthology analysis are represented on the tree. (*B*) We therefore wished to examine potential for our observation of significant apoptosis gene depletion in ecdysozoan genomes to be an artifact of global gene depletion. The distribution of total, nonapoptotic genes (gray) and apoptotic genes (red) that were lost in either Lophotrochozoa (left) or Ecdysozoa (right) are shown. In addition to demonstrating the global trend in gene loss in ecdysozoans as compared with lophotrochozoans, the distributions of apoptotic gene loss in ecdysozoans significantly exceeds that of nonapoptosis genes. We conclude that our finding of apoptosis gene reduction is not an artifact of global genome reduction.

Gene loss through genome reduction has been noted in the evolution of a subset of ecdysozoan genomes included in our study (Copley et al. 2004) and we observe moderate phylogenetic signal (Blomberg’s *K* = 1.07; Pagel’s λ=0.69) in the number of apoptosis genes harbored by taxa in our data set (Keck et al. 2016) (fig. 4*A*). We therefore tested if systematic, global gene loss could drive our observation of widespread depletion of apoptosis genes in ecdysozoans. Although the distributions of genome size and that of apoptosis repertoire size are related (*r*^2^=0.76, *P* ≪ 0.001), apoptosis genes are significantly more depleted in ecdysozoans relative to other genes (Wilcoxon rank-sum *W* = 270,400, *P* = 0.017, fig. 3*B*). Therefore, although ecdysozoan genomes have undergone extensive gene loss, this pattern does not fully explain the significant depletion of apoptosis-related orthogroups in ecdysozoans as we observe more losses of specifically these loci than we would expect by chance.

**Fig. 5.**
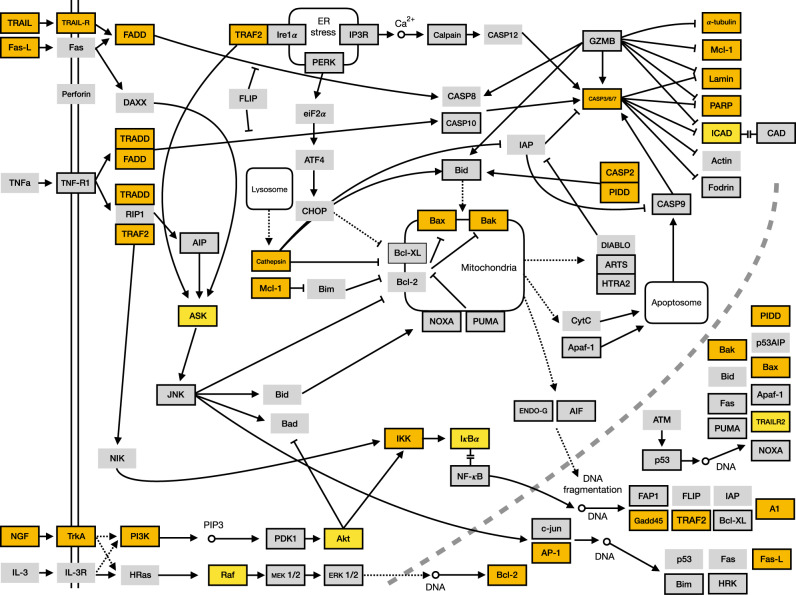
The functional implications of the evolutionary dynamics of the apoptosis gene set. Top. The KEGG apoptosis human pathway is shown redrawn from (Kanehisa et al. 2019). Genes that are significantly depleted in ecdysozoans are highlighted in yellow. This analysis shows that the genes depleted in ecdysozoans function in every aspect of apoptosis including external signaling, mitochondrial disruption, and DNA fragmentation.

Our analyses reveal specific subpathways in the global apoptosis network that have been most affected by the evolutionary dynamics of gene loss during the ecdysozoan radiation. Figure 5 depicts the KEGG apoptosis pathway from mammals (Kanehisa et al. 2019) indicating the broad set of genes that are significantly depleted in ecdysozoan genomes (fig. 3*C*). These ecdysozoan depleted genes function in most known aspects of proapoptotic signaling including the binding of extracellular ligands, mitochondrial disruption, and the DNA damage-mediated response. We interpret this result to suggest that apoptosis-specific gene loss during the ecdysozoan radiation has rendered many of the routes to apoptosis that were present in the bilaterian ancestor unavailable.

### P53 Domain Richness Predicts Apoptotic Gene Repertoire across the Metazoan Tree

**Fig. 6.**
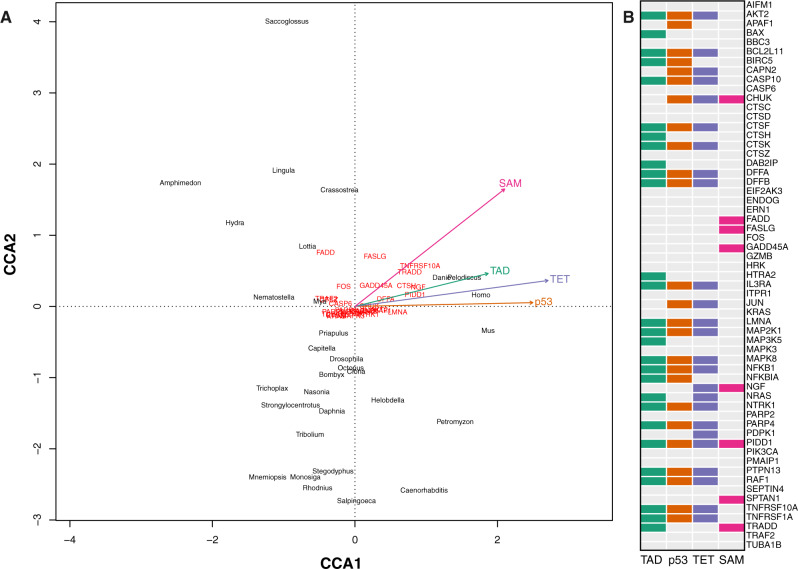
Canonical correspondence and phylogenetic least squares analysis indicate correlations between apoptosis gene loss and p53 domain complexity on the tree. (*A*) Canonical correspondence analysis (CCA) reveals a significant global relationship between apoptosis gene richness and p53 domain richness across taxa (*F* = 2.09, *P* = 0.002). This can be seen by a subset of apoptosis genes (red) pulled in the direction of the p53 domain (TET, p53, TAD, and SAM) ordination vectors. Note the difference between the SAM ordination vector and those of the other domains. The distribution of taxa in Euclidian space as a function of both their apoptotic gene richness and p53 domain complexity is also shown (black). (*B*) We addressed correlations between specific genes and p53 domain richness using phylogenetic least squares analyses. Several domains show overlapping significance with specific genes with the TAD, p53, and TET domains showing the greatest similarity. Colors in (*A*) and (*B*) follow figure 2 (see also supplementary figure 2, Supplementary Material online).

Our results demonstrate that an apoptosis gene repertoire of mammalian complexity was present in the bilaterian ancestor, but extensively remodeled through gene loss in subsequent ecdysozoan lineages. This result mirrors our findings from phylogenetic analyses of p53 and p53 domain structure that showed that the bilaterian ancestor possessed a p53 gene, or genes, with the full complement of ancestral domains, which were later lost in most ecdysozoan lineages. We therefore hypothesized that p53 domain richness would predict apoptosis gene repertoire complexity across metazoan phylogeny. We first examined the relationship between p53 domain richness and apoptotic gene repertoire using canonical correspondence analysis (CCA) (Oksanen et al. 2007). CCA suggests a significant overall association between p53 domain complexity and apoptotic repertoire across taxa (*F*(4,26) = 2.09, *P* = 0.002; fig. 6*A*). We then applied phylogenetic least squares regression (PGLS) (Pinheiro et al. 2019) to determine which p53 domains are predictive of richness for any given apoptosis gene family while accounting for phylogenetic covariance and genome size. We find that the sizes of most apoptosis orthogroups are significantly predicted by the repertoire of one or more specific p53 domains present across the phylogeny (38 out of 59 orthogroups, fig. 6*B*). Here, the per taxon number of TAD, p53, and TET domains largely correlate with a shared set of genes with broad functions across the apoptotic signaling network. Interestingly, the abundance of SAM domains is also correlative with a set of genes in the apoptosis pathway, but these genes show little overlap with the apoptotic gene set predicted by TAD, p53, and TET domain quantity (see also supplementary fig. 2, Supplementary Material online). This suggests that the SAM domain has exerted different effects on the gain and loss dynamics of the apoptotic gene set than that of the TAD, p53, and TET domains. Because p53 is a master regulator of apoptosis in metazoans, we interpret these results to suggest that domain loss in ecdysozoan p53 loci, including the TAD, TET, and SAM domains, has driven wholesale alterations in the structure and function of apoptosis in ecdysozoans, which is evident in reduced distributions of apoptosis genes in extant ecdysozoan genomes.

## Discussion

### The Phylogeny of p53 Like Loci and the Evolutionary Dynamics of Domain Loss

The evolutionary origin of p53 is known to date to at least the last common ancestor of choanoflagellates and metazoans (Belyi et al. 2010) and p53 loci have also been examined within the two major bilaterian lineages of protostomes and deuterostomes (Rutkowski et al. 2010). A clear pattern emerges when the domain structures of p53 are considered across metazoan evolution. First, the p63- and p73-like loci of some lineages, including chordates and lophotrochozoans, are characterized by a complex domain structure that includes the TAD, TET, and SAM domains, in addition to the p53 domain (fig. 2). Further, several lophotrochozoan genomes possess single loci that approximate the additive domain diversity of chordate p53, p63, and p73. In fact, both bivalve species included in the study, *M. arenaria* and the Japanese oyster, *Crassostrea gigas*, possess p53-like loci that include all possible domains (e.g., p53, TAD, TET, and SAM). These observations in chordates and lophotrochozoans sharply contrast with the domain structures observed in nonpriapulid ecdysozoan p53-like loci, which, as per Pfam analyses (El-Gebali et al. 2019) are each devoid of any domain other than the p53 domain itself. The most parsimonious interpretation of these results is that the ancestral bilaterian animal possessed a p53 locus that included the full suite of domains including the TET, TAD, SAM domains, in addition to the p53 domain (fig. 2*B*). This finding leads us to propose that the functional roles of p53-like loci in contemporary ecdysozoan model organisms, including *D. melanogaster* and *C. elegans*, represent a degeneration of ancestral complexity rather than a low-complexity ancestral condition (Lu et al. 2009; Belyi et al. 2010; Fan et al. 2010).

Our phylogenetic analyses also add clarification to a somewhat confusing aspect of p53 gene family nomenclature. Consistent with previous work (Dos Santos et al. 2016), our phylogeny indicates that p53, p63, and p73 clades are each paralogs restricted to the vertebrate lineage. Although the domain structures of several lophotrochozoan sequences are similar to vertebrates p63 and p73 in that they contain TET and SAM domains, they are not directly orthologous to vertebrates p63 and p73 as is often intimated in the literature. Rather, the clade of p53-like proteins in protostomes, including the low-complexity ecdysozoan sequences, is the sister to the vertebrate p53 radiation of genes, which includes p63 and p73. This is an important distinction that precludes p63 and p73 orthologs from existing outside of vertebrates, but they may still serve as functional equivalents. However, our phylogenetic analyses also highlight the challenges of resolving robust topologies for short, highly diverse proteins like p53. Short protein sequences necessarily possess reduced phylogenetic signal and, as we have shown, p53s additionally show highly dynamic evolutionary histories. Therefore, ample caution is required when interpreting our phylogenetic result in figure 2*A*. The positions of the nonpriapulid ecdysozoan p53s in our tree are characteristically toward the root, however, in most cases, there is very poor node support for these positions. Furthermore, the nonpriapulid ecdysozoan p53s fail to form a monophyletic group in our analysis. These factors lead us to question the placement of the nonpriapulid ecdysozoan p53 sequences in our phylogeny (fig. 2*A*), however, we note that none of our central findings is in any way dependent on this result.

### Gene Gain/Loss Dynamics of Apoptosis Loci in Metazoan Genomes Revealed by Orthology Analysis and Dollo Parsimony

p53 proteins are master regulators of apoptosis and modulate several unique signaling cascades that can each lead to apoptosis (fig. 6). The presence or absence of p53 interaction partners in a given genome is one indication of the complexity of the apoptosis pathways that are possible for that taxon. Similarly, the number of proapoptotic loci in a genome has been linked, quantitatively, to apoptotic sensitivity to DNA damage and has been proposed as one possible answer to the incongruence between cancer incidence and organismal cell number, termed “Peto’s paradox” (Peto et al. 1975; Abegglen et al. 2015; Sulak et al. 2016). We hypothesized that the genomes of taxa that have complex p53-like loci would show a quantitative increase in the number of p53-interacting loci that complement and facilitate p53 function in apoptosis. Our orthology analysis demonstrated a pronounced depletion of apoptotic-related loci in ecdysozoan genomes, which also possess low-complexity p53 loci, as compared with chordate and lophotrochozoan genomes. We then confirmed this finding using our implementation of Dollo parsimony which demonstrated that the gene gain and loss dynamics we observed were driven by loss in the ecdysozoan genomes and not gains in chordate or lophotrochozoan genomes (fig. 3*A* and supplementary table 2, Supplementary Material online). Dollo parsimony, where a given trait may originate once on the tree and be freely lost but never regained, has been argued informally in the past to be appropriate for modeling the evolutionary histories of genes and complex traits where the likelihood of re-evolving such traits was considered low (Gould 1993). Although contemporary analyses of trait evolution have demonstrated in fine detail how seemingly homologous complex morphological characters may evolve convergently (Pankey et al. 2014; Sackton and Clark 2019), therefore limiting the suitability of Dollo parsimony for such characters, the model is well suited for genetic loci that have vanishingly low probabilities of evolving independently. Our application of Dollo parsimony to the distributions of p53 domains and apoptosis genes across the metazoan tree provides a stringent assessment of their evolutionary histories while avoiding the need for implementing highly asymmetrical probabilistic models (see Materials and Methods).

### Ecdysozoan Apoptosis Complexity Is Depleted More Than Nonapoptosis Gene Content

We observed a pronounced trend of decreasing apoptosis gene complements among the ecdysozoan genomes utilized in this study (fig. 3). This finding could stem from a global reduction in genes among affected lineages and if so, would not indicate a special property of the ecdysozoan apoptosis gene set. However, we found that the apoptosis gene set is significantly more depleted in ecdysozoan genomes than expected given the global dynamics of reduced ecdysozoan genomic content (fig. 4*B*) suggesting that the ancestral p53-mediated apoptosis gene set of bilaterians was similar in composition to those encoded by modern chordate and lophotrochozoan genomes including *M. arenaria*, but has been subsequently altered by gene loss in Ecdysozoa.

It is interesting to note that molting (ecdysis), a unique and eponymous feature of ecdysozoans, interfaces with apoptosis in this lineage (supplementary fig. 3, Supplementary Material online). We speculate that the origin of molting may have been a proximal factor in the extensive genomic alterations that occurred in both the domain structure of p53 and in the composition of apoptosis pathways during the ecdysozoan radiation, in addition to other profound developmental and life-history consequences that the origin of molting would have required.

### Phylogenetic Correlations between Metazoan p53 Domain Structure and Apoptotic Complexity

We further explored the relationship between p53 domain structure and quantitative aspects of the apoptotic gene set on the tree using global CCA analyses (Oksanen et al. 2007) and phylogenetically informed PGLS (Pinheiro et al. 2019). These analyses lend support for our hypothesis that changes in the domain structure of p53 correlate with changes in gene copy number for apoptosis genes across metazoan phylogeny (fig. 5). The PGLS regression model is especially informative because it accounts for covarying factors such as genome size and the phylogenetic relationships among taxa while assessing the relationship between p53 domain architecture and the repertoire of apoptotic loci. We annotated the human apoptosis pathway based on these analyses and observed that each of the main routes to apoptosis known from mammals are affected either by gene copy number depletion in ecdysozoans and/or compensatory gene gain and loss based on p53 domain structure (fig. 6). In our view, the relationship we observe between the distributions of p53 domains and apoptosis gene copy number suggests a causal mechanism whereby the degeneration of p53 domains releases pleiotropic constraints on loss of other, functionally related apoptotic loci. Furthermore, because the only significant differences in gene number are due to depletion in ecdysozoan linages (fig. 2), we suggest that each of the ancestral pathways to apoptosis, with the exception of the DNA damage-mediated pathway, has been functionally dismantled in the well-studied ecdysozoan models. Support for this hypothesis is given by the fact that only the DNA damage-mediated intrinsic transcriptional pathway to apoptosis has been observed in the ecdysozoans, but other routes to apoptosis including the mitochondrial stress pathway have been observed in nonecdysozoan protostomes including *M. arenaria* (Kelley et al. 2001; Walker et al. 2006).

### Ascertainment Biases Do Not Drive Our Results

Our study design has the potential to be affected by different types of ascertainment biases. The KEGG apoptosis gene set that we chose as the basis for our identification of apoptosis orthogroups represents data drawn from studies in mammalian systems (Kanehisa et al. 2019). Because orthogroup predictions are based in part on BLAST similarity searches (Altschul et al. 1990) the number of apoptosis orthogroups that we recover from a given taxon could be a function of its distance from the mammalian sequences that denote such orthogroups in our analyses. We addressed the potential for artifacts in orthogroup assignment due to this type ascertainment bias in two ways. First, we conducted similar analyses as in figures 3 and 5 using the KEGG apoptosis gene set derived from *Drosophila* data (supplementary fig. 3, Supplementary Material online) (Kanehisa et al. 2019). Our results are clearly similar to those obtained from analyses where mammalian apoptosis sequences were used. We find that among all apoptosis loci considered, the majority of significant quantitative differences in apoptosis gene number are due to depletion in the ecdysozoan lineage, indicating that this general trend is not driven by taxonomic biases. Additionally, due to the stringency of our orthogroup assignment procedure, not all apoptotic loci are recovered in orthogroups in our analyses. If the number of genes recovered in orthogroups was biased by taxon, that is, if some taxa contribute more genes to orthogroups than others, this could induce a different type of ascertainment bias. We tested this by conducting linear regression of the genome-wide number of protein models recovered in orthogroups against the total number of protein models per taxon and find that the two are strongly correlated (*r* = 0.91, *P* value ≪ 0.001, supplementary fig. 4, Supplementary Material online). We conclude that ascertainment biases of this type do not drive our results.

## Conclusions

Understanding the evolutionary history of the metazoan p53-mediated pathway to apoptosis is important if we are to understand how programed cell death has evolved to shape processes of development, immune response, tumor suppression, and the maintenance of multicellularity. The current paradigm in apoptosis research portrays the mammalian pathway as an advanced state that has evolved in complexity to match the organismal complexity of our lineage (Lu et al. 2009). A corollary of the current paradigm is that well-studied ecdysozoan models in apoptosis research (e.g., *D. melanogaster* and *C. elegans*) represent simplified ancestral stock, from which more advanced systems (e.g., mammals) evolved. Here, we applied a diverse data set of phylogenetically informative genome sequences, including the newly sequenced genome of the softshell clam *M. arenaria*, to provide genomic evidence that modulation of apoptosis in lophotrochozoan species is complex and shows many of the same alternative pathways to apoptosis as in mammalian genomes. We also show that, like mammalian genomes, lophotrochozoan genomes are enriched in copy number for several loci involved in apoptosis compared with the genomes of most ecdysozoan species examined. Finally, we note widespread correlations between apoptosis gene copy number and p53 domain complexity on the metazoan tree, indicating a possible mechanism for the observed evolutionary dynamics. Together, our findings suggest that the last common ancestor of bilaterian animals had in place a complex system for apoptosis and that the diversification of bilaterians took place in the context of this extensive apoptotic tool kit. Subsequent alterations of the ecdysozoan apoptosis pathways may have been affected by p53 domain loss and compensatory apoptotic gene reduction trends in these lineages that accompanied the origination of molting, the molecular control of which has co-opted aspects of the apoptosis pathway. Our use of phylogenetic comparative methods illustrates the power of these analyses to elucidate relationships between the elements of multivariate functional genomic data sets and are applicable to a wide range of additional phenotypes (Dunn et al. 2018).

## Materials and Methods

### DNA Extraction and Sequencing

To sequence the genome of *M. arenaria*, we extracted high-molecular weight DNA using the DNAeasy kit (Qiagen) from an individual clam. The resultant DNA was submitted to the Hubbard Center for genomic studies for paired-end Illumina sequencing, where 50× coverage of an estimated 1-Gb genome was performed on a HiSeq 2500 machine. High-molecular weight DNA was extracted from this second individual, and sent to the New York Genome Center for mate pair sequencing where 3- and 7-kb mate pair libraries were prepared for sequencing on the Illumina platform, and to Mount Sinai Medical Center, where low-coverage long-read sequencing was conducted on the Pacific Biosystems RSII platform.

### RNA Extraction, Sequencing, and Assembly

To allow for functional annotation of the newly assembled genome, we extracted RNA using a standard TriZol protocol. We prepared a RNAseq library using the standard Illumina TruSeq protocol, and sequenced it using a paired-end protocol on a HiSeq2500 with reads of length 150 bp. The sequence data were checked for quality using the software FastQC (Andrews 2010) and assembled using the Oyster River Protocol version 2.0 (MacManes 2018).

### Genome Assembly and Evaluation and Annotation

Paired-end libraries were corrected using Lighter (Song and Florea 2015) and assembled using DiscovarDenovo (Weisenfeld et al. 2014) and default settings. The resultant assembly was scaffolded with Soapdenovo (Luo et al. 2012) using the two mate-pair libraries. The scaffolds were gap filled with PBJelly (English et al. 2012) using the low-coverage PacBio data. The resultant genome was evaluated using the various methods. First, the contiguity of the genome was evaluated using the software package Quast (Gurevich et al. 2013), using default settings. Next, the genic contiguity was evaluated using the software BUSCO version 3.0.2 (Simao et al. 2015), which was run in genome mode against the eukaryotic database. The final genome was annotated using the software Maker, version 3.0.0 (Cantarel et al. 2007). The two ab initio genome prediction tools, SNAP (Korf 2004) and Augustus (Stanke and Waack 2003) were trained using the output from BRAKER (Hoff et al. 2016) and BUSCO (Simao et al. 2015), respectively. In addition to this, the transcriptome assembly was used to provide functional data related to gene structure. All sequence data are available at Sequence Read Archive accession PRJEB33745.

### Generation of Choanozoan p53 Data Set

We conducted BlastP (Altschul et al. 1990) searches of known p53 orthologs (*Mus musculus* AAA39883.1 and *Drosophila* NP_996267.1) against the protein models from a selection of 28 whole-genome sequences representing choanoflagellates, ctenophores, poriferans, cnidarians, placozoans, and bilaterians (supplementary table 3, Supplementary Material online). BLAST searches were conducted with an expect value of 0.001 and all sequences meeting this threshold were retained (Shah et al. 2019). Hits from each taxon search were combined and redundant sequences were removed using cdhit (Li and Godzik 2006). The procedure resulted in a total of 90 putative p53 orthologs. All alignments were done with MAFFT (Katoh et al. 2009) under the *linsi* setting. After the initial alignment, eight sequences that had incomplete p53 DNA binding domains were removed from the data set and the data set was realigned as before. Gaps in the resulting alignment that included fewer than 5% sequence overlap across the alignment were then removed using trimal (Capella-Gutierrez et al. 2009). Concurrently, we assessed the protein domain structure for all sequences in our final data set using a batch search of the Pfam data set using HMMER3 (El-Gebali et al. 2019) and an expect value of 0.001. Inspection of the alignment and the Pfam results showed that all sequences possessed a p53 binding domain and most also included the p53 TET (fig. 1*A* and *B*). The resulting alignment included 82 protein sequences and 845 positions with gaps (supplementary data set 1, Supplementary Material online). All bioinformatic, phylogenetic, and analytical codes are provided at https://github.com/plachetzki/p53_clam.git

### Phylogenetic Analysis of the P53 Gene Family

Initial phylogenetic analyses were done using IQ-Tree V 1.6.2 (Chernomor et al. 2016) under the -MFP option that searches for the best-fit model (VT+F+R6) from a large number of possibilities. Nodal support was assessed by approximate likelihood ratio scores (alrt) (Anisimova and Gascuel 2006) and ultrafast boostrapping (Hoang et al. 2018). Phylogenetic alignment plots were done using the R package ggtree2 (Yu et al. 2018). Domain structures were plotted using custom scripts and the R packages phytools (Revell 2012) and ape (Paradis 2018).

### Orthology Analyses of Metazoan Protein-Coding Genes

We conducted an orthology analysis of all protein models from the 29 whole-genome sequences included in the study, including the protein models from the genome of the soft-shelled clam *M. arenaria* using Orthofinder (Emms and Kelly 2015). Our analyses resulted in 29333 orthogroups with 6991 orthogroups having representation for at least 50% of the taxa. We obtained the human sequences associated with the KEGG apoptosis gene set (Kanehisa et al. 2019) which we used to identify 59 apoptosis-related orthogroups, based on their cross-referenced human sequences. We then tabulated the genes placed in each orthogroup, apoptosis, or otherwise, for each taxon. These data were used to determine clade-wise enrichment among 1) apoptosis orthogroups and for 2) all other orthogroups. Clade-wise enrichment/depletion analyses were conducted using the Fisher‐Pitman permutation test in R (R Core Team 2013). Phylogenetic heatmaps were constructed using phytools (Revell 2012). The effect of genome size on apoptosis gene repertoire was measured with linear regression (supplementary fig. 4, Supplementary Material online). PGLS further tested this relationship while including phylogeny as covariate with apoptosis gene sets evolving under Brownian motion (Pinheiro et al. 2019). Relative depletion of apoptotic repertoire was quantified by taking the natural log of the ratio of orthogroup counts in Lophotrochozoa+Deuterostomia to those in Ecdysozoa. Differences in the ratios between apoptotic orthogroups and nonapoptotic orthogroups were tested with the Wilcoxon rank-sum test. Tests for phylogenetic signal were conducted using the R package phylosignal (Keck et al. 2016). Ancestral predictions for apoptotic repertoire and p53 domain structure were estimated under Dollo parsimony using functions from the R pacakages “phytools” and “phangorn” (Schliep 2011; Revell 2012). The effect of p53 domain complexity on apoptosis orthogroup sizes was assessed using CCA function “cca” from the R package “vegan” (Oksanen et al. 2007). The effect of individual p53 domain counts on apoptotic orthogroup size was then quantified using a generalized least squares model along with genome size and including a correlation structure of apoptosis gene set evolving under Brownian motion on the phylogeny. 

## Supplementary Material

evaa143_Supplementary_Data

## Notes

**Data deposition:** All sequence data are deposited at Sequence Read Archive under the accession PRJEB33745.
