# Supplementary material for: The Genome of the Softshell Clam Mya arenaria and the Evolution of Apoptosis
Source: Genome Biol Evol. 2020 Jul 11;12(10):1681–93. doi: 10.1093/gbe/evaa143 (PMC7531772; doi:10.1093/gbe/evaa143)
Supplement: evaa143_Supplementary_Data [file evaa143_supplementary_data.pdf]

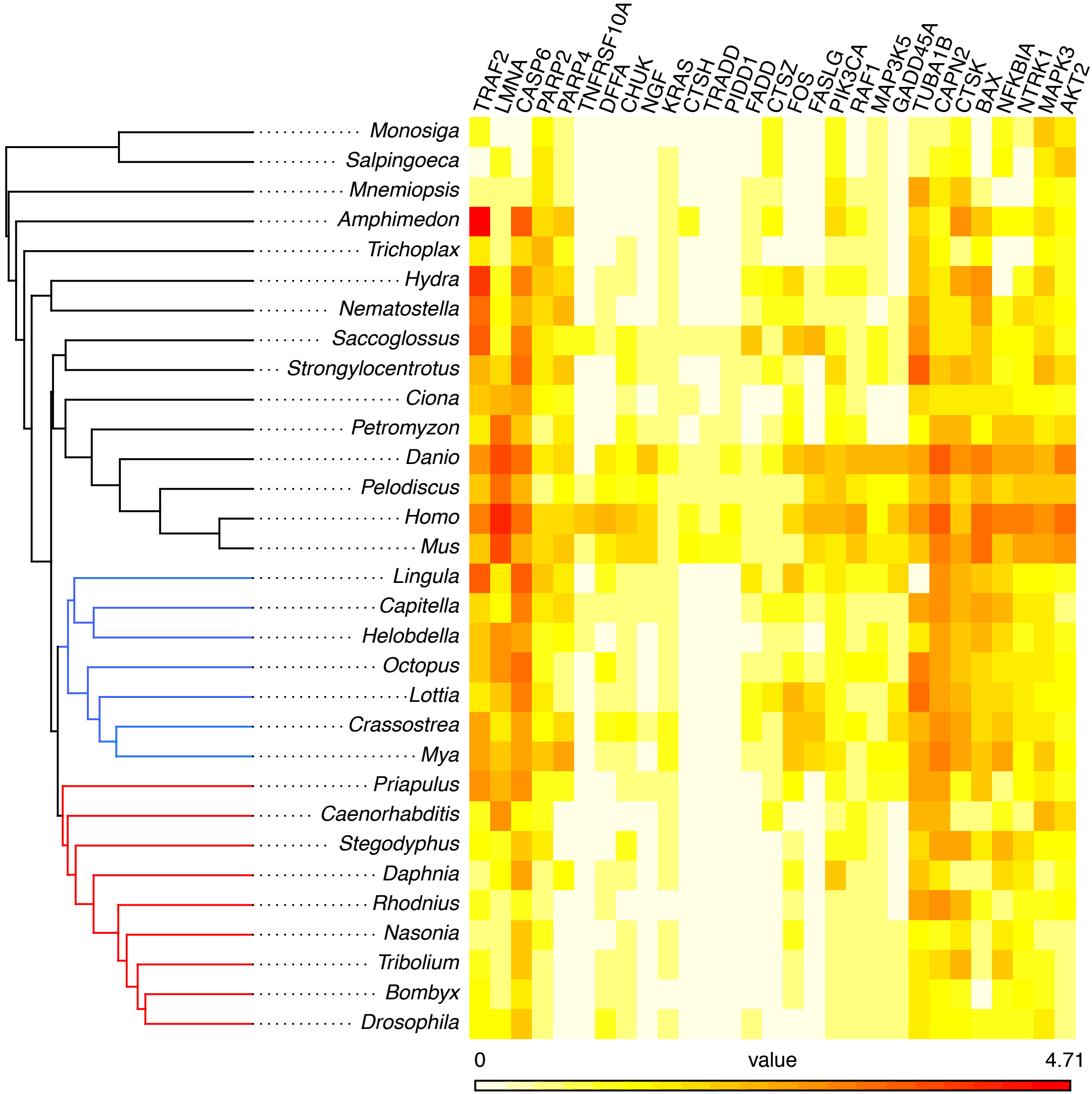

Supplementary Figure 1

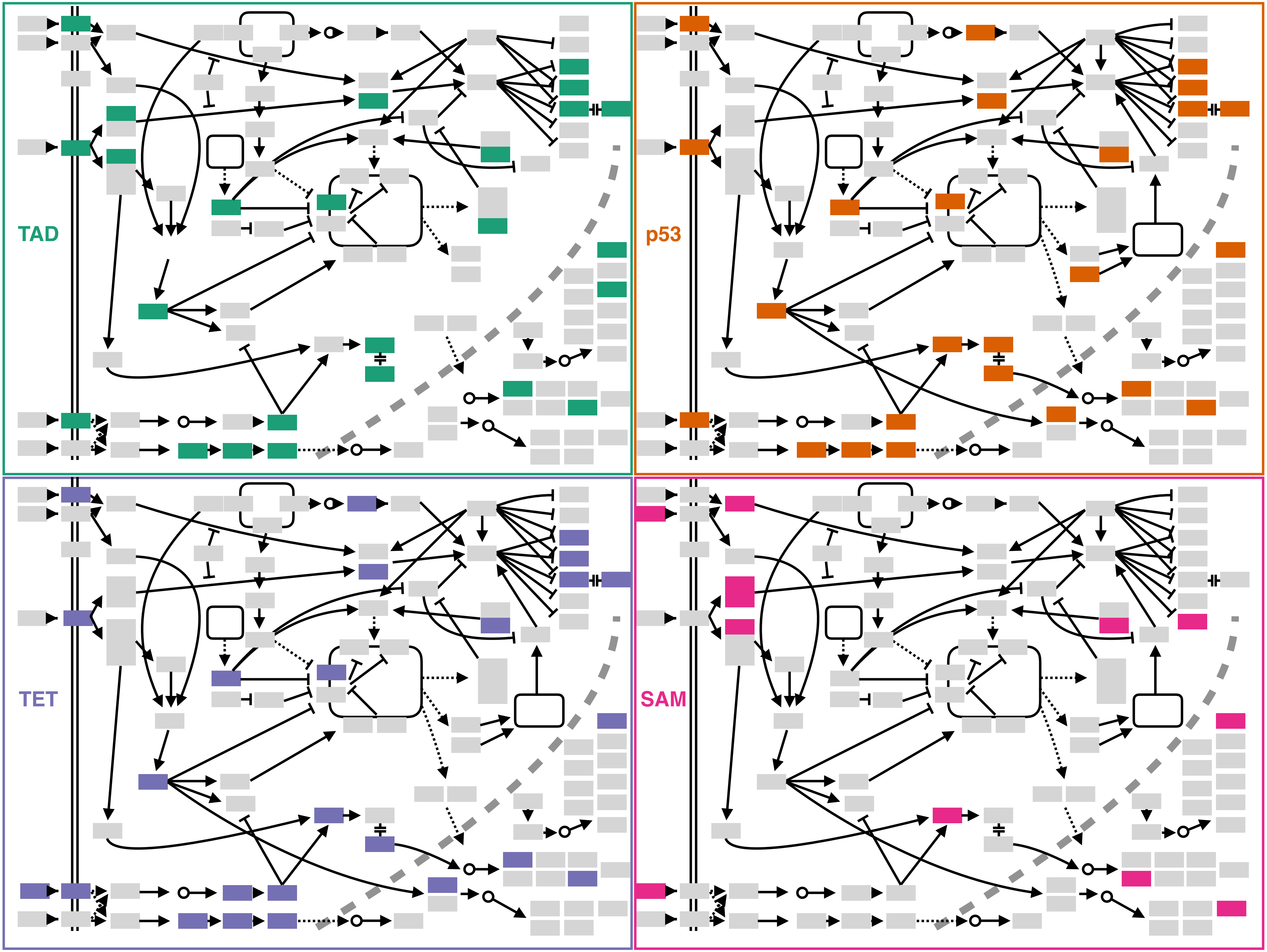

Supplementary Figure 2

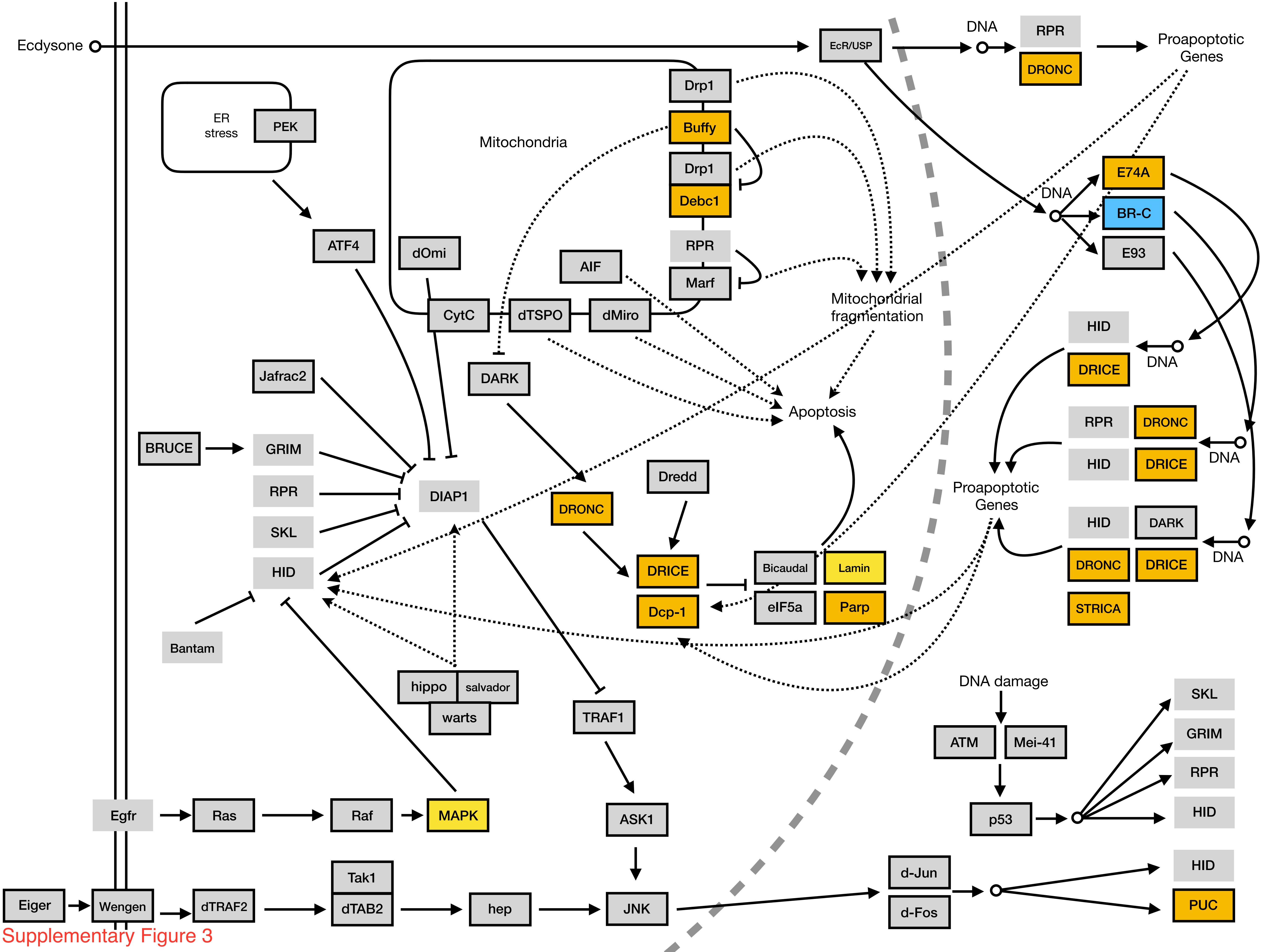

Supplementary Figure 3

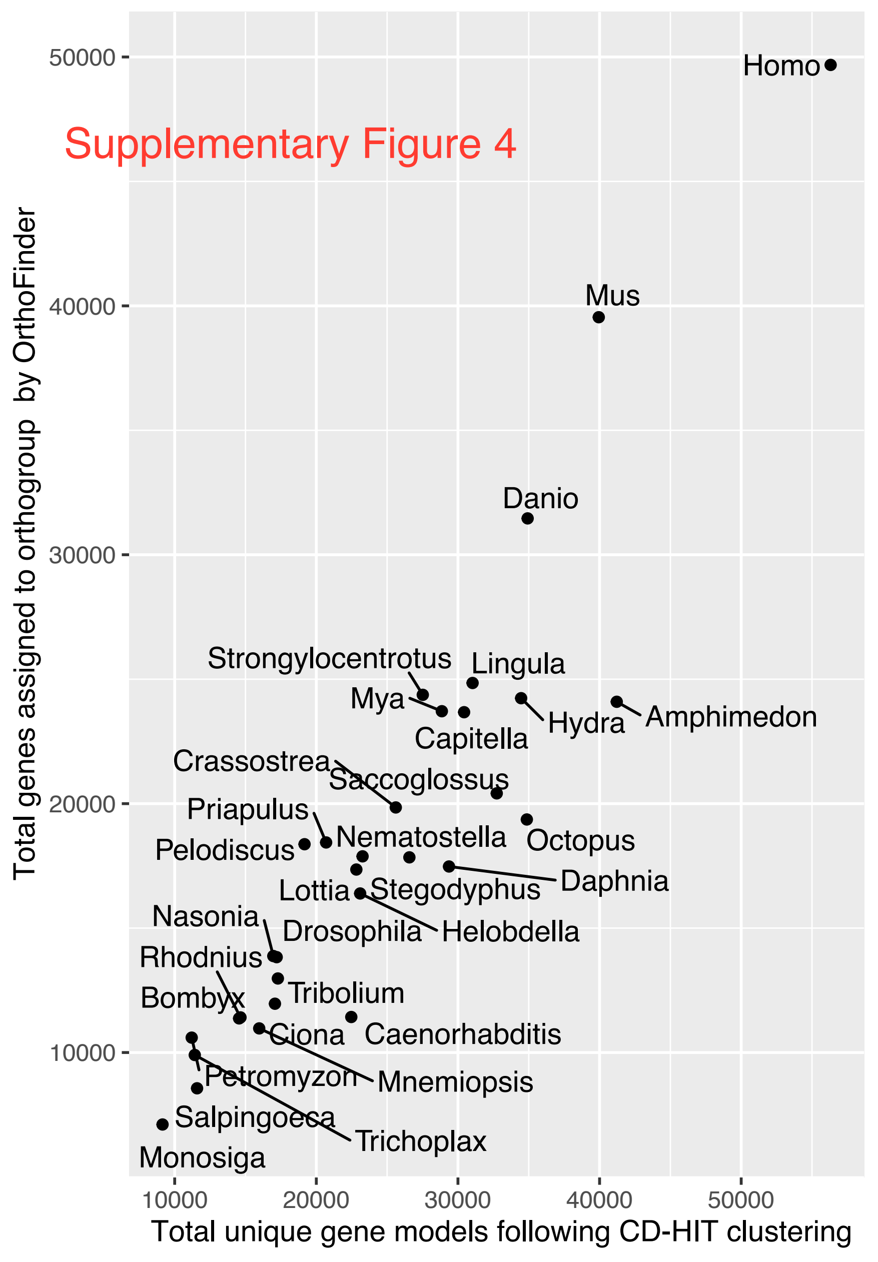
Supplementary Figure 4
Total genes assigned to orthogroup by OrthoFinder
Total unique gene models following CD-HIT clustering
50000
40000
30000
20000
10000
10000
20000
30000
40000
50000
Homo
Mus
Danio
Strongylocentrotus
Lingula
Mya
Hydra
Amphimedon
Capitella
Crassostrea
Saccoglossus
Priapulus
Nematostella
Octopus
Pelodiscus
Daphnia
Lottia
Stegodyphus
Nasonia
Drosophila
Helobdella
Rhodnius
Tribolium
Bombyx
Ciona
Caenorhabditis
Petromyzon
Mnemiopsis
Salpingoeca
Trichoplax
Monosiga

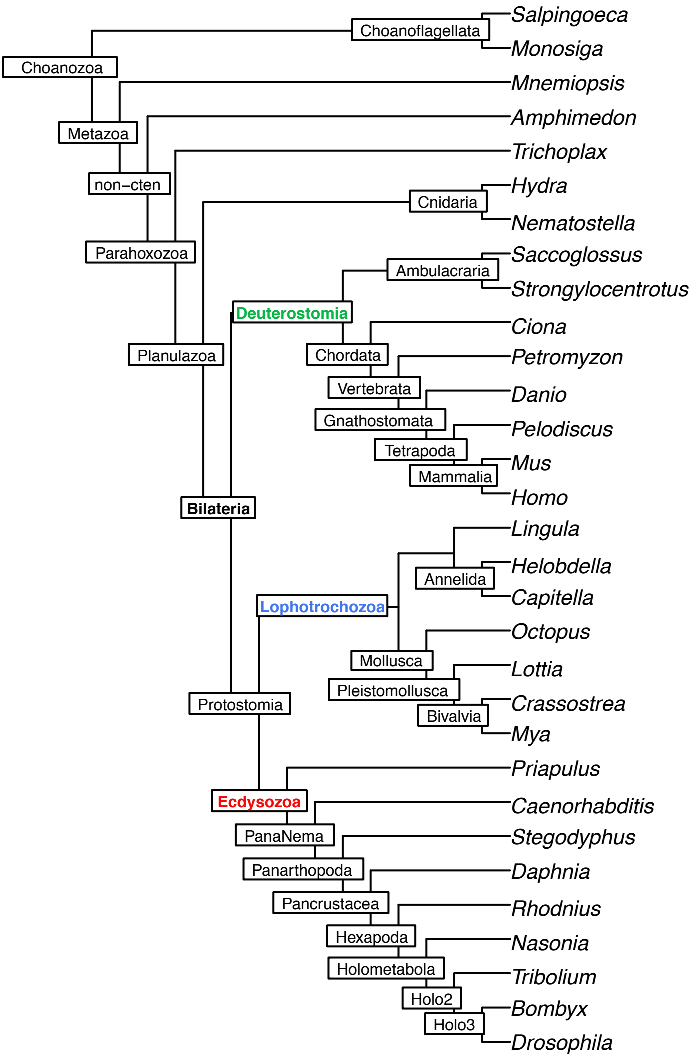

Supplementary Figure 5
